# Examining whether affectively-charged motivations predict subsequent affective response during physical activity: An ecological momentary assessment study

**DOI:** 10.3389/fspor.2022.1029144

**Published:** 2022-11-18

**Authors:** Bridgette Do, Ryan E. Rhodes, Martina Kanning, Micaela Hewus, Genevieve F. Dunton

**Affiliations:** ^1^Department of Population and Public Health Sciences, Keck School of Medicine, University of Southern California, Los Angeles, CA, United States; ^2^School of Exercise Science, Physical and Health Education, University of Victoria, Victoria, BC, Canada; ^3^Department of Sports Science, Social and Health Sciences, University of Konstanz, Konstanz, Germany; ^4^Department of Psychology, University of Southern California, Los Angeles, CA, United States

**Keywords:** affective, physical activity, exercise, motivation, experience sampling method (ESM)/ecological momentary assessment (EMA)

## Abstract

**Background:**

Evidence suggests positive affective response during physical activity increases the likelihood of engaging in and maintaining regular activity exercise in the future. Elucidating antecedents for a positive affective response may help identify intervention strategies to increase activity. Affectively-charged motivations (e.g., desires, urges, dreading) have been posited as proximal antecedents to physical activity but have yet to be examined in terms of their influence on affective response in real-world settings. The current study used ecological momentary assessment (EMA) to examine within-subject effects of pre-physical activity affectively-charged motivation on subsequent affective response during physical activity.

**Methods:**

Participants included 56 adults (*M* = 39.18 years, *SD* = 11.98; 67.86% female) who completed a 14-day smartphone-based EMA study. Prior to starting physical activity (time *t*), participants self-initiated an event-contingent EMA survey that assessed affectively-charged motivation for physical activity (i.e., rating scale from “dreading it” to “excited to do it”). EMA surveys prompted during subsequent physical activity (time *t* + 15 min) assessed affective response (i.e., feeling good—bad, energized—exhausted, thrilled—miserable, interested—bored, and relaxed—nervous). Multi-level linear regression models examined within-subject effects of pre- physical activity affectively-charged motivations on subsequent affective response during physical activity controlling for between-subjects effects of affectively-charged motivation, age, biological sex, time of day, and day of the week.

**Results:**

Overall, there were *N* = 304 physical activity occasions in the analysis (*M* = 5.43, *SD* = 3.97). When individuals reported more positive affectively-charged motivation for physical activity than usual before physical activity occasions, they reported feeling more energized (Estimate = 0.22, *p* < 0.001), good (Estimate = 0.25, *p* < 0.001), thrilled (Estimate = 0.12, *p* = 0.02), and interested (Estimate = 0.24, *p* < 0.001) during subsequent physical activity. Affectively-charged motivation was not associated with feeling more relaxed (Estimate = 0.11, *p* = 0.13) during subsequent physical activity.

**Conclusion:**

Momentary affectively-charged motivations predicted more positive affective response during subsequent physical activity among active adults. Future research can explore potential sources of intraindividual differences in affectively-charged motivations and further examine these associations with future physical activity behavior. To improve positive affective responses, interventions may boost affectively-charged motivations through real-time mobile prompting in naturalistic settings.

## Introduction

Affective constructs have received increased attention and have been applied to conceptual models (1–4), theories, research, and interventions for physical activity (5–7). Consistent with hedonic theories of behavior, affective response during physical activity has been posited as a key determinant of future physical activity behavior (8). Hedonic theories suggest humans tend to maximize pleasure and minimize displeasure, and affective response to a behavior may influence future decisions on whether or not to repeat that behavior (9, 10). Affective response during physical activity has been shown to be associated with future activity levels; research suggests positive affective response during physical activity increases the likelihood of engaging and maintaining physical activity in the future (11). For example, adults who engaged more positive affective response during a moderate-intensity bout of exercise in the laboratory reported more minutes of physical activity at both 6 and 12 months later (12). In practice, these findings translate to a one-unit increase in positive affective response on an 11-point scale being associated with an increase in 38 and 41 min of physical activity per week at 6 and 12 months, respectively (12). The World Health Organization recommends adults engage in at least 150 min of moderate-to-vigorous-intensity physical activity per week for optimal health benefits such as reducing the risk for cardiovascular and metabolic diseases, some cancers, symptoms of depression and anxiety, and premature mortality (13). However, most adults do not meet the recommended levels of activity (14). Therefore, understanding the antecedents to having a positive affective response during physical activity can help identify intervention strategies to promote regular physical activity.

Affectively-charged motivations have been posited as proximal antecedents to physical activity but have yet to be examined in terms of their relevance to affective response to physical activity. While there are varying conceptualizations of motivations for physical activity (4, 15), Stevens and colleagues state affectively-charged motivation for physical activity includes motivational states that have their basis in past affective responses to physical activity, such as hedonic motivation (i.e., desire, dread, craving), intrinsic motivation, and fear (8). Affectively-charged motivation is related to affective response such that people typically crave or desire experiences in which they have had a previous positive affective response (4). Affectively-charged motivation and affective response may be based in hedonism, but they are two distinct constructs that may individually and uniquely contribute to physical activity. It is also unclear whether affectively-charged motivation predicts how one will respond affectively when they engage in subsequent physical activity. Elucidating the proximal antecedents of affective response is critical for understanding how to improve affective response, and in turn increase physical activity behaviors.

Previous literature has examined concurrent determinants of affective response to physical activity. For example, higher positive affective response was reported when engaging in physical activity with other people as compared to being alone, and higher negative affective response was reported when engaging in physical activity indoors as compared to outdoors (16). Robust findings from laboratory studies have also demonstrated the association between physical activity intensity and affective response (17). Findings indicate homogenous negative shifts in affective valence as physical activity intensity increases beyond one's ventilatory threshold, yet there is heterogeneity in affective response to moderate-intensity activity based on fitness levels, body composition, and cognitive factors (18, 19). Despite these findings, there is limited research exploring the interconnection between affective constructs.

Previous research on affective response to physical activity has largely been conducted in laboratory settings; however, research in naturalistic settings is necessary to elucidate these processes in naturally-occurring physical activity in everyday life and how these processes occur on a *moment-to-moment* basis. Ecological momentary assessment (EMA)—a real-time data capture methodology that involves repeatedly assessing participants' current states, behaviors, and context in naturalistic settings—is well-suited to gather data on naturally occurring phenomenon such as affective constructs (20). EMA methods aim to reduce biases related to recall by assessing current states and behaviors, or recent ones. Unlike traditional retrospective study designs, EMA methods can provide information at the momentary level. Additionally, this data can more accurately portray participants' typical context and settings (21). Multiple assessments through EMA can also address research questions about within-person variation in affective determinants and how processes unfold over time. For example, within-person variability in desires, cravings, and wants have been less explored. Results across five validation studies for the Cravings for Rest and Volitional Energy Expenditure scale indicated desires/wants are transient and more of a motivation state rather than a trait (22). Understanding whether there are intraindividual differences in affectively-charged motivation on affective response during physical activity can highlight the importance of targeting affectively-charged motivation in interventions.

Therefore, the objective of the current study was to use EMA to examine the within-subject effects of pre-physical activity affectively-charged motivation on subsequent affective response during physical activity in naturalistic settings among physically active adults. It was hypothesized that on physical activity occasions when individuals reported more pre-physical activity affectively-charged motivation than usual, they would report more positive affective response during subsequent physical activity. Specifically, reporting feeling more excited (vs. dread)—than usual—before a physical activity bout would be associated with reports of feeling more energized, good, thrilled, interested, and relaxed during subsequent physical activity. Feeling good (vs. bad), energized (vs. exhausted), thrilled (vs. miserable), interested (vs. bored), and relaxed (vs. nervous) were selected to assess affective response in order to represent the two fundamental dimensions of affect as suggested by the circumplex model (i.e., valence and arousal) (23).

## Materials and methods

### Overview and design

An intensive longitudinal study design using a 14-day interval-contingent/fixed time-based and event-contingent EMA study examined affective mechanisms associated with recreational, leisure-time physical activity. Data collection took place between August 2020 and August 2021. All study recruitment and data collection took place remotely to eliminate in-person interactions during the coronavirus disease 2019 (COVID-19) pandemic. This study was conducted in accordance with the Declaration of Helsinki and all aspects of the study were approved by the Institutional Review Board at the University of Southern California (UP-20-00321).

### Participants and recruitment

To be eligible for the study, participants needed to: (1) be between the ages of 18–65 years old; (2) able to read and speak in English; (3) use an Android smartphone as their primary personal phone; and (4) currently engage in at least 60 min of structured exercise a week. Exclusion criteria included: (1) cardiovascular, respiratory, muscular, or bone/joint problems that preclude physical activity; (2) taking psychotropic medications; (3) receiving treatment for any psychiatric disorder; (4) inability to answer smartphone-based survey for extending periods of time due to work, care giving, or driving requirements; (5) have a body mass index under 18.5 kg/m^2^ or over 50 kg/m^2^ (categorized as underweight or morbidly obese); (6) current cigarette smoker; (7) diabetic; (8) unable or unwilling to answer EMA surveys while exercising; (9) been treated for cancer within the past 6 months (e.g., chemotherapy, surgery, radiation); or 10) swim for exercise more than once a week. Inclusion and exclusion criteria were established to: include participants that regularly engage in physical activity to guarantee activity data in naturalistic settings; consider the health and safety of participants; ensure that sufficient data is collected from the smartphone during the 14-day period.

Potential participants were recruited through social media postings, website postings, and advertisements on platforms such as Facebook, Twitter, Instagram, and Reddit. Emails were also sent to potentially eligible participants through ResearchMatch (a national health volunteer registry that was created by several academic institutions and supported by the U.S. National Institutes of Health as part of the Clinical Translational Science Award program). Previous study participants from University of Southern California research studies—who had previously indicated that they were willing to be contacted for future research—were also contacted through email. Interested individuals were asked to complete an electronic interest form, in which study staff assessed for initial eligibility. If participants were considered potentially eligible, they were then immediately prompted to fill out a short questionnaire to further assess eligibility. Eligible participants were then contacted by a study staff member to participate in the study.

### Procedures

Eligible participants were invited to complete a baseline session through video conference with study staff. During the baseline session informed consent was obtained and a consent form was electronically signed. Participants were given a link to complete an electronic baseline questionnaire though an online survey platform REDCap (Research Electronic Data Capture) (24). Participants were able to complete the survey on a mobile phone, tablet, or desktop device. Participants received instructions on how to download the study smartphone application on the participant's personal smartphone and answer EMA surveys through a virtual orientation with a study staff member. Participants also wore a waist-worn accelerometer during the 14-day period; however, the data was not used in the current analyses to answer the study objectives. After the study period, participants completed an exit interview through videoconference with a study staff member. Participants received up to 150 USD for study participation with incentives based upon compliance to study procedures.

### Measures

#### Ecological momentary assessment

Participants downloaded a free EMA application and completed 14 consecutive days of EMA on their personal Android smartphone. EMA data were collected through a commercial software mobile phone application for Android smartphones (movisensXS by movisens GmbH; Karlsruhe, Germany). Participants responded to event-contingent EMA surveys. Participants were instructed to manually initiate a “pre- physical activity” EMA survey in the smartphone application before each time that they did physical activity during the 14-day period. The survey took about 1 min to complete. Following the completion of the pre- physical activity EMA survey, participants were instructed to begin their physical activity as planned. The smartphone application automatically prompted additional “during- physical activity” EMA surveys 15 min after the completion of the pre- physical activity survey through an audible noise and or/vibration. Participants were asked to briefly pause their activity, complete the EMA survey if it was safe to do so, and then return to their activity. Each during- physical activity survey took about 30 s to complete. If no entry was made, the application sent one reminder signal in 5 min. After the reminder signal, the EMA survey closed and was no longer accessible to the participant.

#### Affectively-charged motivations

During the pre-physical activity EMA surveys participants were asked “Do you intend to exercise in the next 15 min?” with answer options “Yes,” “No,” “Not sure.” If “yes” was selected, participants were subsequently asked “How do you feel about exercising in the next 15 min?” A sliding scale from “Dreading it” to “Excited to do it” was provided on the screen. Although participants were not presented numeric values on the screen, the EMA application assigned a numeric value to where the slider was positioned ranging from 0 (“Dreading it”) to 100 (“Excited to do it”); higher values indicated more positive affectively-charged motivation. This item was developed by the study investigators/authors.

#### Pre-physical activity affect

The pre-physical activity EMA prompt assessed momentary affect. Participants were asked “How do you feel right now?” On the same screen, five visual analog scales were presented: “Bad” to “Good,” “Exhausted” to “Energized,” “Miserable” to “Thrilled,” “Bored” to “Interested,” and “Relaxed” to “Nervous” (23, 25–27). A numeric value, ranging from 0 to 100, was assigned to the slider position. For the analyses, higher values indicated feeling more good, energized, thrilled, interested, and relaxed.

#### Affective response

The during-physical activity EMA prompt assessed affective response during physical activity. The first item of the survey asked, “Are you finished exercising?” If participants indicated “No,” then affective response during physical activity was measured. Participants were asked “How do you feel right now?” On the same screen, five visual analog scales were presented: “Bad” to “Good,” “Exhausted” to “Energized,” “Miserable” to “Thrilled,” “Bored” to “Interested,” and “Relaxed” to “Nervous” (23, 25–27). Each sliding scale did not have visible numeric values; however, the EMA application assigned a numeric value based on the slider position ranging from 0 to 100, with higher values indicating feeling more good, energized, thrilled, interested, and nervous. For the analyses, feeling nervous was reverse coded so a higher value indicated feeling more relaxed.

#### Participant characteristics

Participants self-reported the following characteristics in the baseline survey: age, sex at birth (male, female), gender identity (male, female, trans male/trans man, trans female/trans woman, genderqueer/gender non-conforming, different identity) race (American Indian or Alaska Native, Asian Indian, Black or African American, Chinese, Filipino, Guamanian or Chamorro, Japanese, Korean, Native Hawaiian, Other Asian, Other Pacific Islander, Samoan, Vietnamese, and White); ethnicity (Hispanic, Latino/a, or Spanish origin, not Hispanic, Latino/a, or Spanish origin); total combined family income for the past 12 months (< $12,000, $12,000–24,999, $25,000–34,999, $35,999–44,999, $45,000–54,999, $55,000–64,999, $65,000–74,999, $75,000–84,999, $85,000–94,999, $95,000–104,999, $105,000–114,999, $115,000–124,999, and >$125,000); and employment status (employed for wages, self-employed, out of work for 1 year or more, out of work for < 1 year, homemaker, student, retired, and unable to work). Participants were not required to answer these questions if they preferred not to.

### Statistical analyses

The analytic plan was specified prior to the analysis. To test the study objective of examining within-subject effects of pre-physical activity affectively-charged motivation on subsequent affective response during physical activity, multi-level linear regression models were conducted. Affectively-charged motivation was disaggregated into within-subject (Level-1, physical activity occasion) and between-subject (Level-2, participant) levels to partition the variance (28). The within-subject variance represents the deviation from one's own mean on any given physical activity occasion and the between-subject variance represents the individual mean deviation from the grand mean deviation (mean of all observations across all participants) (29). The outcomes—feeling good, energized, thrilled, interested, and relaxed—were examined in separate multi-level models. All models adjusted for the following variables a priori: between-subject effects of affectively-charged motivation, pre-physical activity affect, age, sex at birth (female, male), time of day (morning 6 A.M.-12 P.M.; afternoon 12 P.M.-6 P.M.; evening 6 P.M.-6 A.M.), day of week (weekday, weekend day). All analyses were conducted in SPSS Version 25 and statistical significance was set at *p* < 0.05. Unstandardized regression coefficients, standard errors, and p-values are reported.

## Results

### Data availability

A total of 720 individuals expressed interest in the study and completed the online eligibility questionnaire. Of this number, 206 individuals were eligible, and 76 individuals subsequently agreed to participate in the study and completed informed consent. A total of 68 participants had some completed EMA data (i.e., responded to at least one EMA survey) during the 14-day period. There were 684 completed pre- physical activity observations and 327 completed during- physical activity observations (i.e., 15 min after the completion of the pre- physical activity EMA prompt) among 66 participants. After matching the pre- physical activity and during- physical activity observations, the final analytic sample included *N* = 56 participants (Level-2) and *N* = 304 physical activity occasions (Level-1).

### Participant characteristics

Descriptive statistics for participant characteristics are shown in Table 1. Participants ranged in age from 22 to 65 years with an average age of 39.2 (*SD* = 12.0) years. Most participants selected “female” sex at birth and self-identified as White. About 64% of the sample were employed for wages and 43% reported a total combined family income for the past 12 months of >$65,000. The average number of physical activity occasions per participant was 5.43 (range 1–19) during the 14-day period. The mean affectively-charged motivation [0 (“Dreading it”) to 100 (“Excited to do it”)] was 75.2 (*SD* = 20.2). Affective response during physical activity scores were: feeling good (M = 77.7, *SD* = 14.4), feeling energized (M = 72.7, *SD* = 17.7), feeling thrilled (M = 71.8, *SD* = 15.4), feeling interested (M = 73.7, *SD* = 17.5), and feeling relaxed (M = 71.1, *SD* = 19.9).

**Table 1:** Participant characteristics (*N* = 56).

**Demographics**	***n* (%)**
**Age in years (Mean** **±SD)**	39.2 ± 12.0
**Sex at birth**	
Female	38 (67.9)
Male	18 (32.1)
**Gender identity**	
Female	36 (64.3)
Male	18 (32.1)
Trans female/trans woman	1 (1.8)
Trans male/trans man	0 (0)
Genderqueer/gender non-conforming	1 (1.8)
**Hispanic, Latino/a, or Spanish origin**	
Yes	5 (8.9)
No	51 (91.9)
**Race^a^**	
American Indian or Alaska Native	2
Asian Indian	6
Black or African American	9
Chinese	2
Filipino	1
Guamanian or Chamorro	0
Japanese	0
Korean	2
Other Asian	1
Other Pacific Islander	0
Native Hawaiian	0
Samoan	0
Vietnamese	1
White	36
**Education**	
High school graduate	3 (5.4)
Some college or technical school	6 (10.7)
College graduate	47 (83.9)
**Work status**	
Employed for wages	36 (64.3)
Self-employed	5 (8.9)
Out of work for 1 year or more	2 (3.6)
Out of work for < 1 year	3 (5.4)
Homemaker	3 (5.4)
Student	6 (10.7)
Retired	1 (1.8)
**Annual household income^b^**	
< $35,000	14 (25.5)
$35,000–64,999	17 (30.9)
$65,000–114,999	16 (29.1)
≥ $115,000	8 (14.5)

a Participants were able to select all that apply.

b Data missing for one participant.

### Within-subject effects of affectively-charged motivation on affective response during physical activity

A series of multi-level linear regressions examined the within-subject effects of affectively-charged motivation on affective response during subsequent physical activity. Unstandardized coefficients and standard errors are shown in Tables 2–4. Neither day of week (weekend day vs. weekday) or time of day (morning vs. evening and afternoon vs. evening) were significantly associated with affective response in all four models. There were significant within-subject effects such that when individuals reported more positive affectively-charged motivation for physical activity than usual before physical activity occasions, they reported feeling more energized (Estimate = 0.22, *p* < 0.001), good (Estimate = 0.25, *p* < 0.001), thrilled (Estimate = 0.12, *p* = 0.02), and interested (Estimate = 0.24, *p* < 0.001) during subsequent physical activity. Results indicate that for every 10-point increase in affectively-charged motivation (i.e., excited to do it vs. dreading it), their affective response (i.e., feeling energized, good, thrilled, and interested) during subsequent physical activity score increased by about 2 points. Affectively-charged motivation was not associated with feeling more relaxed (Estimate = 0.11, *p* = 0.13) during subsequent physical activity.

**Table 2:** Multi-level models examining the association between affectively-charged motivation and feeling good and feeling energized.

	**Good-bad**		**Energized-exhausted**	
	**Estimate (*SE*)**	***p***	**Estimate (*SE*)**	***p***
Intercept	77.66 (2.99)	< 0.001	65.29 (5.74)	< 0.001
Level-1 (Physical activity bout)				
Within-subject affectively-charged motivation	0.25 (0.04)	< 0.001	0.22 (0.07)	< 0.01
Within-subject pre-physical activity affect^a^	0.15 (0.05)	< 0.01	0.09 (0.05)	0.09
Weekday^b^	0.44 (1.07)	0.68	0.09 (1.67)	0.99
Time of day (morning)^c^	0.81 (1.44)	0.58	4.43 (2.41)	0.07
Time of day (afternoon)^d^	0.23 (1.42)	0.87	0.23 (2.34)	0.92
Level-2 (Person)				
Between-subject affectively-charged motivation	0.22 (0.07)	< 0.01	0.38 (0.09)	< 0.001
Between-subject pre-physical activity affect^a^	0.65 (0.09)	< 0.001	0.45 (0.13)	< 0.01
Age	−0.05 (0.07)	0.48	0.11 (0.13)	0.41
Sex at birth (female)^e^	4.27 (1.61)	0.01	1.01 (3.21)	0.75

N = 56 participants, N = 304 day-level observations.

a Corresponding pre-physical activity affect (i.e., good or energized).

b Weekday vs. weekend day.

c Morning (6 A.M.−12 P.M.) vs. evening (5 P.M.−6 A.M.).

d Afternoon (12 P.M. −5 P.M.) vs. evening (5 P.M. −6 A.M.).

e Female vs. male.

**Table 3:** Multi-level models examining the association between affectively-charged motivation and feeling thrilled and feeling interested.

	**Thrilled-miserable**		**Interested-bored**	
	**Estimate (*SE*)**	***p***	**Estimate (*SE*)**	***p***
Intercept	67.69 (4.08)	< 0.001	71.04 (4.07)	< 0.001
Level-1 (Physical activity bout)				
Within-subject affectively-charged motivation	0.12 (0.05)	0.02	0.24 (0.05)	< 0.001
Within-subject pre-physical activity affect^a^	0.29 (0.05)	< 0.001	0.20 0.05)	< 0.001
Weekday^b^	−0.53 (1.27)	0.67	0.54 (1.34)	0.69
Time of day (morning)^c^	2.68 (1.78)	0.13	1.06 (1.87)	0.57
Time of day (afternoon)^d^	2.22 (1.73)	0.20	0.77 (1.82)	0.67
Level-2 (Person)				
Between-subject affectively-charged motivation	0.23 (0.08)	< 0.01	0.19 (0.08)	0.02
Between-subject pre-physical activity affect^a^	0.60 (0.10)	< 0.001	0.71 (0.09)	< 0.001
Age	0.04 (0.09)	0.67	0.02 (0.09)	0.80
Sex at birth (female)^e^	3.02 (2.21)	0.18	1.53(2.22)	0.50

N = 56 participants, N = 304 day-level observations.

a Corresponding pre-physical activity affect (i.e., thrilled or interested).

b Weekday vs. weekend day.

c Morning (6 A.M.−12 P.M.) vs. evening (5 P.M.−6 A.M.).

d Afternoon (12 P.M.−5 P.M.) vs. evening (5 P.M.−6 A.M.).

e Female vs. male.

**Table 4:** Multi-level models examining the association between affectively-charged motivation and feeling relaxed.

	**Relaxed-nervous**	
	**Estimate (*SE*)**	***p***
Intercept	77.79 (5.03)	< 0.001
Level-1 (Physical activity bout)		
Within-subject affectively-charged motivation	0.11 (0.07)	0.13
Within-subject pre-physical activity affect^a^	0.42 (0.06)	< 0.001
Weekday^b^	−1.82 (1.87)	0.33
Time of day (morning)^c^	−0.42 (2.49)	0.87
Time of day (afternoon)^d^	−1.63 (2.45)	0.51
Level-2 (Person)		
Between-subject affectively-charged motivation	0.15 (0.08)	0.05
Between-subject pre-physical activity affect^a^	0.68 (0.08)	< 0.001
Age	−0.12 (0.11)	0.24
Sex at birth (female)^e^	−0.37 (2.68)	0.89

N = 56 participants, N = 304 day-level observations.

a Corresponding pre-physical activity affect (i.e., relaxed).

b Weekday vs. weekend day.

c Morning (6 A.M.−12 P.M.) vs. evening (5 P.M.−6 A.M.).

d Afternoon (12 P.M.−5 P.M.) vs. evening (5 P.M.−6 A.M.).

e Female vs. male.

## Discussion

To our best knowledge, the current study is the first known attempt to assess associations between affectively-charged motivation and subsequent affective response during physical activity in naturalistic settings among physically active adults. Furthermore, EMA data was used to examine interindividual differences in affectively-charged motivations (i.e., within-subject effects) on affective response. Findings indicated greater momentary affectively-charged motivation predicted more positive affective response during subsequent physical activity. On physical activity occasions when individuals reported more positive affectively-charged motivation for physical activity than usual, they reported feeling more energized, good, thrilled, and interested, but not more relaxed, during subsequent physical activity. This study contributes knowledge on proximal antecedents of affective response to physical activity and underscores the importance of examining how variations in motivation for physical activity works in the moment.

Despite the recent call to attention for examining motivation states for movement (15), this area of research has been mostly overlooked. A recently published scoping review protocol on urges to move and motivation states for physical activity among healthy and clinical populations noted that the scope of the literature describing affectively-charged motivational states remains largely unknown (30). Little research has been conducted on measuring desire or craving for physical activity; the vast majority of research on motivation states focused on health behaviors such as alcohol, smoking, substance use, and eating (31–33). However, one published study investigated desires for experiences people's natural environments and findings revealed desire strength was significantly above average for sports participation, and other experiences including sleep, sex, hygiene, social contact, and non-alcoholic drinks (34). Our study's findings contribute to the limited literature base on affectively-charged motivation for physical activity and provide further evidence for motivation states varying from moment-to-moment. Future studies can extend these findings by conducting additional intensive longitudinal studies on affectively-charged motivations in naturalistic settings among different populations and examine potential associations with other affective determinants (e.g., processed affect) and objectively-measured activity.

Given the previous evidence for associations between affective response and future physical activity behaviors (10, 11), intervention strategies which result in positive shifts in affective valence during physical activity need to be developed. The current study's findings suggest that efforts to influence affective response during physical activity may be more effective if affectively-charged motivations are considered or targeted. Stults and colleagues proposed general approaches for increasing desires or urges to move, such as: modifying the reward value of exercise by making it more pleasurable/less punishable, environmental and situational conditions to boost motivation states to move, ‘nudging' people in response to desires/cravings, and taking advantage of urges/desires for other rewarding behaviors to encourage the development of desires to move (15). An example of the latter approach is the gamification of Pokémon GO—which is contingent on physical movement—and therefore may increase the reward value of movement (35). Real-time mobile prompting may also prove to be efficacious for increasing physical activity by targeting momentary affectively-charged motivations in real-time and real-world settings. Given that recommendations have also been applied to affective processing, additional research on distinct techniques for targeting affectively-charged is warranted (7).

Our findings provide support for the associations between affectively-charged motivation and affective response (i.e., feeling good, energized, thrilled, and interested) during physical activity, but associations with feeling relaxed were non-significant. The null associations between affectively-charged motivation and feeling relaxed may be due to the fact that affectively-charged motivation (“feeling excited”) is an activating motivation, whereas “feeling relaxed” is a deactivated affective response. While both items are similar in valence (i.e., positive), they differ in their activation state. To build upon these findings, additional research is needed to examine whether these associations predict future physical activity behaviors. Longitudinal research with repeated assessments or experimental designs could elucidate the potential causal pathways in which affectively-charged motivation and affective response influence physical activity (8). Furthermore, studies could determine whether solely targeting affectively-charged motivation or solely targeting affective response, or both simultaneously, is the most successful for promoting future activity. Some intervention strategies—such as evaluative conditioning (i.e., changing attitudes through an object's pairing with a positively valanced stimuli)—may influence multiple affective constructs such as affectively-charged motivation, affective response, and affective processing (7, 8, 36). Considering multiple affective determinants and how they vary from moment-to-moment can strengthen previous physical activity interventions predominantly focused on targeting cognitive factors.

### Strengths and limitations

The study had several strengths such as the assessment of multiple affective constructs in naturalistic settings and the ability to examine within-subjects associations through multiple assessments per participant. To our best knowledge, this is the first published study to examine associations of affectively-charged motivations and affective response through EMA in participants' everyday lives. The study extends previous research conducted in laboratory settings, yielding more generalizable findings. However, the study did have some limitations. First, only one item was used to assess affectively-charged motivation for physical activity in the pre-physical activity EMA survey. Future EMA studies could use or adapt items from newer measurement scales; in example, the Cravings for Rest and Volitional Energy Expenditure (CRAVE) assessment measures motivation states (i.e., wants, desires, urges) for physical activity and sedentary behavior “at this very moment (i.e., Right now)” (22). Prior to the development of CRAVE, there has not been a validated instrument to assess affectively-charged motivation specifically to physical activity and previous research often asked participants to retrospectively report their motivations. Future EMA research could incorporate validated measures, and future research needs to seek a consensus on terms and definitions used to describe the constructs of desire, craving, wanting, and dread. Furthermore, conceptual and theoretical models can establish whether these are each unique, individual constructs in regards to the target behavior, or if they fall under distinguished categories such as affectively-charged motivational states, hedonic motivation, or automatic motivation (15). Another study limitation to note is that the study may not have captured all physical activity occasions that occurred during the 14-day study period. Participants were given the option to dismiss the during-physical activity EMA survey prompt, and they may have dismissed a survey if it was unsafe for them to complete the survey while exercising (e.g., biking, running through city streets). One of the study's strengths was the ability to assess self-initiated physical activity occurring in naturalistic settings. However, it may not be feasible or safe to capture all types of physical activity. Lastly, the sample largely consisted of self-identifying females from middle-to-higher income households, who also were already engaging in regular physical activity (i.e., at least 60 min per week). Findings may not be generalizable to other sub-groups such as those who are not currently physically active. In addition, the analytic sample included 56 adults (73% of participants enrolled in the study). However, the intensive longitudinal study design allowed us to examine over 300 physical activity occasions. Additional research is needed to examine these momentary associations in larger, more representative samples.

## Conclusion

Overall, study findings suggest momentary affectively-charged motivation predicted more positive affective response during subsequent physical activity in naturalistic settings among active adults. These results contribute to the limited literature on affectively-charged motivation for physical activity and underscore the importance of including affective constructs in research and physical activity programming. Future studies should examine casual pathways in which affectively-charged motivation and affective response predict future engagement in physical activity. Interventions seeking to promote physical activity may benefit by developing targeted strategies to enhance affectively-charged motivation.

## Data availability statement

The raw data supporting the conclusions of this article will be made available by the authors, without undue reservation.

## Ethics statement

The studies involving human participants were reviewed and approved by University of Southern California Institutional Review Board. The patients/participants provided their written informed consent to participate in this study.

## Author contributions

BD wrote the first draft of the manuscript and performed the statistical analysis. BD, GD, and MH contributed to the conception and design of the study. BD, RR, MK, MH, and GD contributed to manuscript editing and revision. All authors read and approved the submitted version.

## Funding

Funding for this research was provided by a University of Southern California Zumberge Large Interdisciplinary Grant and by the National Institutes of Health, National Heart, Lung, and Blood Institute (3U01HL146327-03S1). This work was also supported by the Achievement Rewards for College Scientists Foundation Los Angeles Founder Chapter.

## Conflict of interest

The authors declare that the research was conducted in the absence of any commercial or financial relationships that could be construed as a potential conflict of interest.

## Publisher's note

All claims expressed in this article are solely those of the authors and do not necessarily represent those of their affiliated organizations, or those of the publisher, the editors and the reviewers. Any product that may be evaluated in this article, or claim that may be made by its manufacturer, is not guaranteed or endorsed by the publisher.
